# Evaluation of Calcium Electroporation for the Treatment of Cutaneous Metastases: A Double Blinded Randomised Controlled Phase II Trial

**DOI:** 10.3390/cancers12010179

**Published:** 2020-01-10

**Authors:** Dóra Ágoston, Eszter Baltás, Henriette Ócsai, Sándor Rátkai, Péter Gy Lázár, Irma Korom, Erika Varga, István Balázs Németh, Éva Dósa-Rácz Viharosné, Julie Gehl, Judit Oláh, Lajos Kemény, Erika Gabriella Kis

**Affiliations:** 1 Department of Dermatology and Allergology, University of Szeged, 6720 Szeged, Hungary; agoston.dora@med.u-szeged.hu (D.Á.); baltas.eszter@med.u-szeged.hu (E.B.); ocsai.henriette@med.u-szeged.hu (H.Ó.); dr.ratkai.sandor@gmail.com (S.R.); korom.irma@med.u-szeged.hu (I.K.); varga.erika@med.u-szeged.hu (E.V.); nemeth.istvan.balazs@med.u-szeged.hu (I.B.N.); viharosne.eva@med.u-szeged.hu (É.D.-R.V.); kemeny.lajos@med.u-szeged.hu (L.K.); 2 Department of Oral and Maxillofacial Surgery, University of Szeged, 6720 Szeged, Hungary; lazarpgy@gmail.com; 3 Center for Experimental Drug and Gene Electrotransfer (C*EDGE), Department of Clinical Oncology and Palliative Care, Zealand University Hospital, 4000 Roskilde, Denmark; kgeh@regionsjaelland.dk; 4 Department of Clinical Medicine, Faculty of Health and Medical Sciences, University of Copenhagen, 2200 Copenhagen, Denmark; 5 Department of Oncotherapy, University of Szeged, 6720 Szeged, Hungary; lazarne.olah.judit@med.u-szeged.hu

**Keywords:** calcium electroporation, bleomycin-based electrochemotherapy, cutaneous metastases, melanoma malignum, breast cancer, randomization, biopsy, non-inferiority

## Abstract

Calcium electroporation (Ca-EP) is a new anticancer treatment providing similar features to electrochemotherapy (ECT). The aim of our study is to compare the efficacy of Ca-EP with bleomycin-based ECT. This double-blinded randomized controlled phase II study was conducted at the Medical University of Szeged, Hungary. During this once only treatment up to ten measurable cutaneous metastases per patient were separately block randomized for intratumoral delivery of either calcium or bleomycin, which was followed by reversible electroporation. Tumour response was evaluated clinically and histologically six months after treatment. (ClinicalTrials.gov: NCT03628417, closed). Seven patients with 44 metastases (34 from malignant melanoma, 10 from breast cancer) were included in the study. Eleven metastases were taken for biopsies, and 33 metastases were randomised and treated once. The objective response rates were 33% (6/18) for Ca-EP and 53% (8/15) for bleomycin-based ECT, with 22% (4/18) and 40% (6/15) complete response rates, respectively. The CR was confirmed histologically in both arms. Serious adverse events were not registered. Ulceration and hyperpigmentation, both CTCA criteria grade I side effects, were observed more frequently after bleomycin-based ECT than for Ca-EP. Ca-EP was non-inferior to ECT, therefore, it should be considered as a feasible, effective and safe treatment option.

## 1. Introduction

Bleomycin-based ECT is a widely used method for the treatment of cutaneous tumours from all histologies [1,2,3,4,5,6]. During ECT, a chemotherapeutic drug, usually bleomycin, is electroporated into the tumour cells, resulting in an increased cytotoxic effect. A recent meta-analysis of ECT in a palliative setting found a complete response (CR) rate of 46.6% and objective response rates (ORR) of 82.2%, regardless of the tumour type. Beyond its effectiveness, ECT is a repeatable and minimally invasive intervention that reduces symptom burden [7].

With the goal of reducing the risk of possible adverse events, the chemotherapeutic drug (bleomycin) is replaced with calcium in calcium electroporation (Ca-EP). Notably, calcium might be an effective option in cases where the administration of a chemotherapeutic drug is contraindicated. It was also expected that the use of Ca-EP will simplify the procedure and lower the treatment cost.

The mechanism of action and the anticancer efficacy of Ca-EP have been confirmed in preclinical studies. During Ca-EP treatment, electroporation increases intracellular calcium concentration, leading to increased ATP consumption. Additionally, the treatment leads to further loss of ATP production by opening permeability transition pores in mitochondrial membranes. This ATP depletion, together with other cellular effects, causes cell death [8].

The features of Ca-EP are very similar to ECT: they are both selective to tumour cell, the same pulsing conditions can be used for both procedures, and both have an anti-angiogenic effect in vitro and in vivo on both normal and tumour blood vessels [9,10]. It has also been demonstrated that Ca-EP initiates a systemic immune response. Thus, Ca-EP has the potential to replace bleomycin with calcium in electroporation treatments.

The first clinical trial of Ca-EP in humans was published recently [11]. The method was used with seven patients (six with breast cancer, one with malignant melanoma), and safety and non-inferiority in comparison with bleomycin-based ECT was proven.

To provide additional evidence on the efficacy and safety of Ca-EP, we decided to conduct a clinical trial. The aim of this study is to evaluate safety and efficacy of Ca-EP and to compare it with bleomycin-based ECT for cutaneous metastases.

## 2. Materials and Methods

This non-inferiority, phase II, double blinded, randomized confirmatory study investigated the efficacy of Ca-EP in comparison with the currently approved bleomycin-based ECT procedure for the treatment of cutaneous metastases. The study protocol was authorized by the Health Registration and Training Centre on 3 May 2016 and registered under the case number 032104/2016/OTIG. It was approved by the national and institutional ethical review boards (SZTE Regional Institutional Ethics Committee (23 May 2016, license no. 3806; registry no. 98/2016-SZTE). Patients provided written informed consent before enrolment.

The primary endpoint of the study was to compare the tumour response of cutaneous metastases after application of Ca-EP and ECT with the administration of intratumoral bleomycin. Tumour response was evaluated similar to response evaluation criteria in solid tumours (RECIST), v1.1, on each treated metastasis by clinical examination and digital photo documentation before and after treatment. The secondary endpoint was to evaluate and grade the toxicity of Ca-EP similar to Common Terminology Criteria for Adverse Events (CTCAE), version 4.0. The third endpoint was the measurement of the maximum safe and effective delivery of current to metastases using Ca-EP and bleomycin-based ECT.

Patients enrolled in the study had at least one histologically confirmed metastasis of 0.5 to 3 cm in size that was accessible to electroporation. A maximum of 10 cutaneous metastases per patient was included in the trial. Depending on the number of metastases present on the patient, one to six metastases were randomized into one of the two treatments and to the right or the left arm for evaluating response. Calcium was administered intratumorally to tumour(s) of one arm and bleomycin to the other and administration was immediately followed by electroporation of tumours on both arms.

Patient inclusion criteria were as follows: age >18 years, World Health Organization (WHO) performance status ≤2, life expectancy more than 3 months, platelet count ≥50 billion/L, international normalised ratio (INR) <1.5, and a period of more than 2 weeks of without treatment [12]. Only medical cancer treatments (endocrine treatment, targeted treatment and radiotherapy to another area) were allowed. If there was no regression of cutaneous metastases, the continuation of vinorelbine, capecitabine or paclitaxel therapies were allowed. Patients were excluded from the trial if they had severe allergic reactions associated with bleomycin or if they previously received a dose of bleomycin that was more than 200,000 units/m². Pregnancy and lactation were also reasons for exclusion. Previously irradiated cutaneous metastases and concomitant treatments were recorded. The intervention was performed after oncological recommendation.

### 2.1. Randomization and Blinding

A clinical pharmacologist, who was independent of all the parties concerned with the study, was in charge of randomisation and kept the randomisation list secure, with the task of providing limited access only in case of emergency. The numbered metastases in each patient were block randomised 1:1 separately into the two treatment arms with the nQuery Adviser 7.0 computer program. The drugs to be given for each metastasis were prepared and labelled by a clinical pharmacologist. There was no need to cover the content of the ready syringes, as both bleomycin and calcium-chloride are colourless.

### 2.2. Procedure

The concentration of calcium chloride was estimated to be 220 mmol/L (9 mg/mL) from preclinical studies [9,12,13,14] and of bleomycin was 1000 IU/mL.

The volume to be injected was calculated according to the volume of the tumour. The drug volume for large tumours (>0.5 cm^3^) was 0.5 mL/cm^3^, while for small tumours (<0.5 cm^3^) the volume was amended to 1 mL/cm^3^. Tumour volume was calculated as ab2π/6, where a = longest diameter, b = longest diameter perpendicular to a.

Electric pulses were generated using a Cliniporator device (IGEA, Carpy, Italy) according to the standard operating procedures of the electrochemotherapy (ESOPE) guidelines. Linear needle electrodes (8 pulses of 400 V and 1000 V/cm, of 0.1 ms duration, at a frequency of 5 kHz) and hexagonal needle electrodes (4 pulses of 730 V and 910 V/cm, of 0.1 ms duration, at a frequency of 5 kHz) were used according to the tumour size and location. The anaesthesia during the procedure was either local or general, as planned during the consultation between the physician and the patient. Lidocaine with epinephrine was used as local anaesthetic and was injected in a square around the nodule. Changes in the response to anaesthesia were not observed.

Patients underwent the treatment once and were followed for 12 months with scheduled visits (7, 15, 30, 60, 90, 180 and 360 days after the treatment session).

At each follow up visit, the tumour response was evaluated clinically, and photos were taken. Response was categorised according to criteria similar to RECIST guidelines 1.1 as follows: complete response (CR), disappearance of all target lesions; partial response (PR), at least a 30% decrease in the sum of the longest diameter of target lesions; stabile disease (SD), neither sufficient shrinkage to qualify for PR nor sufficient increase to qualify for progression; and progression of the disease (PD), at least a 20% increase in the sum of the longest diameter of target lesions [15].

The randomization code was revealed 6 months after treatment and biopsies were taken from both calcium- and bleomycin-treated lesions. All biopsies were analysed by a histopathologist for the amount of tumour tissue, inflammation, fibrosis and necrosis.

Safety was evaluated with physical examinations and blood tests before and after treatment. Quality of life (QOL) score (0–100%) was also evaluated before and after the treatment. A numeric rating scale (NRS) (0–10) was used for assessing pain [16]. The Common Toxicity Criteria for Adverse Events, version 4.0, was used to register possible adverse events [17].

#### All Participants Assessed the Primary and Safety Analyses

The statistical analyses were performed using IBM SPSS, v24, software and R statistical program. Tumour response was analysed using Fisher’s exact test on objective response 6 months after treatment, Mann-Whitney test was used to measure the difference in delivered current between calcium-EP and bleomycin-based ECT, and the 2-sided 95% CI was used to measure the difference in outcome between the two groups [18]. For dimensioning the required number of cutaneous metastases, a non-inferiority study analysis was used: calculated with a significance level of 0.05 and a power of 80%, the results indicated that a minimum of 28 evaluable tumours were needed [19]. Taking into consideration the results of the previous clinical trial for the treatment of Ca-EP on cutaneous metastases as well as the basis of preclinical studies, a 20% non-inferiority margin was preset to detect a clinical difference between the two treatment arms.

## 3. Results

**Figure 1:**
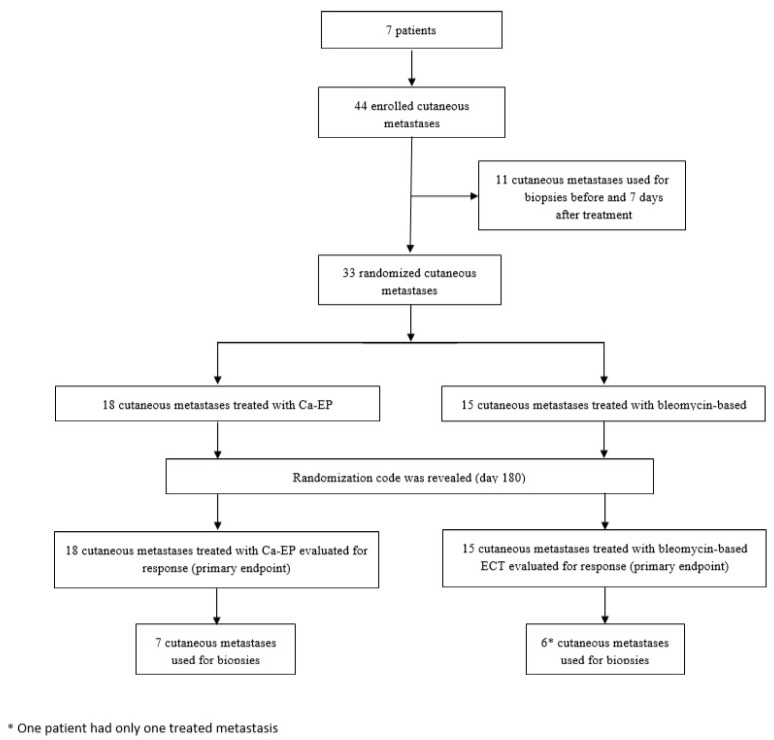
Trial profile. Illustration of trial profile. Further results are described in detail.

**Figure 2:**
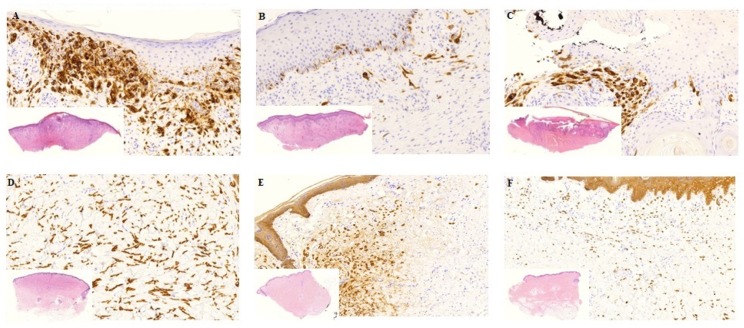
Histology from biopsies before and 7 days after treatment. Biopsies before and one week after treatment. (**A**–**C**) Patient nr. 5 with MM. A: Pre-treatment biopsy: Extensive tumour infiltration, mild fibrosis, moderate lymphocytic inflammation, no necrosis. The tumour cells show diffuse MelanA positivity. (**B**) Day 7. Post-treatment with Ca-EP: Partly ulcerated skin, moderate tumour infiltration and fibrosis, mild inflammation, no necrosis. Only scattered MelanA positive tumour cells. (**C**) Day 7. Post treatment with bleomycin-based ECT: Partly fragmented, ulcerated skin with pseudoepitheliomatous hyperplasia of the epidermis, moderate fibrosis and inflammation, no necrosis. Focal MM nests with MelanA positivity. (**D**–**F**) Patient nr. 2 with breast cancer. (**D**) Pre-treatment biopsy: Extensive breast cancer infiltration without fibrosis, inflammation and necrosis. The tumour cells are CKAE1/AE3 positive. (**E**) Day 7. Post-treatment with Ca-EP: Focal tumour infiltration, very mild inflammation no fibrosis or necrosis. (**F**) Day 7. Post treatment with bleomycin-based ECT: Dispersed tumour cells with CKAE1/AE3 positivity, mild inflammation, no fibrosis or necrosis. MM: malignant melanoma, CK: cytokeratin. Histological photos: digital scanning with magnification approximately 5–20×.

Seven patients (5 females, 2 males) with a total of 44 cutaneous metastases (34 from melanoma malignum and 10 from breast cancer) were enrolled in the clinical trial between October 2016 and June 2018 (Figure 1). Six patients had cutaneous metastases of malignant melanoma, localized on the lower extremity and one patient had metastases of breast cancer, localized on the trunk (Table 1). The patients median age was 73 years (Interquartile range: IQR = 21). Thirty-three metastases were randomized into the two treatment arms (left or right) of the study and were evaluated for clinical response, whereas 11 lesions were taken as biopsies (Figure 2). Eighteen of the randomized metastases were treated with Ca-EP (15 melanoma malignum cutan metastases, 3 breast cancer metastases) and 15 with bleomycin-based ECT (12 melanoma malignum cutan metastases, 3 breast cancer metastases). Six (18%) of the 33 randomized cutaneous metastases were located on a previously irradiated area (2 lesions were treated with Ca-EP, and 4 lesions with bleomycin-based ECT). According to the 33 response-evaluated metastases, the median was 7 mm (IQR = 5) of the largest diameters. The median injected volume for Ca-EP was 0.0855 mL (IQR = 0.1924), and 0.132 mL (IQR = 0.27) for bleomycin-based ECT (Table 2).

Four of the procedures were conducted under local, and three of them under general anaesthesia. Hexagonal needle electrodes were used for electroporation of 21 metastases, (63.6%), whereas linear electrodes were used for 12 lesions (36.4%). Of the 18 lesions receiving Ca-EP, 11 were treated with hexagonal (61.1%) and 7 with linear electrodes (38.9%), respectively. Of the 15 lesions receiving bleomycin-based ECT, 10 were treated with hexagonal electrodes (66.7%), and 5 with linear electrodes (33.3%).

### 3.1. Tumor Response

**Figure 3:**
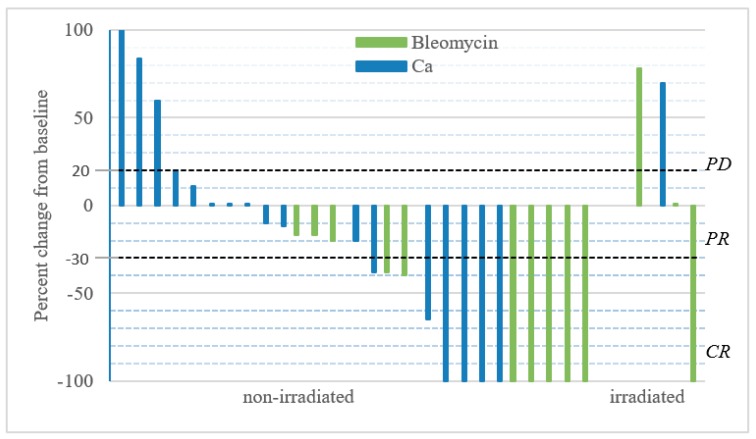
Change in tumour size over time. Metastases were treated at day = 0 with either i.t. calcium and or i.t. bleomycin in a randomized double-blinded study design. Patients received only one treatment and response was evaluated 6 months after treatment, after the randomization code was revealed. Change in size over time; the graph illustrates the percent change in tumour size recorded 6 months after treatment. The two non-measurable metastases treated with calcium-chloride and bleomycin were irradiated, and are not included in the graph, but were included in the response analysis as PD.

**Figure 4:**
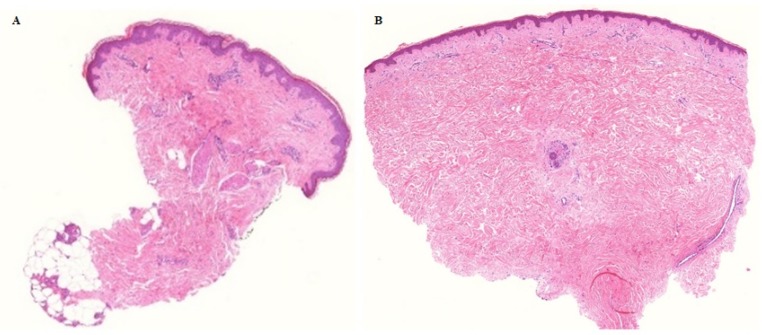
Histologically confirmed complete remission 6 months after Ca-EP. Tumour cells were not identified 6 months after Ca-EP neither in malignant melanoma (**A**) nor in breast cancer metastases (**B**). A: patient no 3, Ca-EP treated clinically CR melanoma malignum cutaneous metastasis. B: patient no 2, Ca-EP treated clinically CR breast cancer cutaneous metastasis. Histological photos: digital scanning with low magnification.

The ORR for Ca-EP was 33% (CR = 22%; PR = 11%) and for bleomycin-based ECT was 53% (CR = 40%; PR = 13%) (Figure 3). The difference was not significant neither in OR (*p* = 0.30) nor in CR (*p* = 0.45) between Ca-EP and bleomycin-based ECT. After 6 months, 33% (6 of 18) metastases treated with calcium, and 13% (2 of 15) metastases treated with bleomycin had progressed. The two-sided 95% CI for the outcome difference between the two groups was −13.3%–53.3%. There was no significant difference in response between previously irradiated and non-irradiated lesions (*p* = 0.37). Six months after treatment, the randomization code was revealed, and 6 biopsies (3 from Ca-EP, 3 from bleomycin-treated lesions) taken from the 13 tumours exhibited a clinically CR. For 5 of these 6 biopsies, a CR was confirmed by histology (Figure 4). No tumour cells were identified in the 3 lesions treated with Ca-EP and in 2 of the lesions treated with bleomycin-based ECT.

Regarding tumour response and the type of electrode, we observed differences between the two treatment arms that because of the small sample size was statistically not significant. The ORRs obtained with Ca-EP cutaneous metastases were higher for linear electrodes (42.8%, 3/7) than for hexagonal electrodes (27.3%, 3/11) (*p* = 0.63). Of the tumours that were categorized as PD, 45.45% (5/11) were treated with hexagonal and 14.29% (1/7) with linear electrodes (*p* = 0.32). The opposite trend was observed with bleomycin-based ECT. The ORR was 70% (7/10) with hexagonal and 20% (1/5) with linear electrodes (*p* = 0.12). Of the tumours that were categorized as PD, 10% (1/10) were treated with hexagonal and 20% (1/5) with linear electrodes (*p* = 1).

Six patients had long-term follow-up over a mean of 29 months (standard deviation: σ = 6.8232). One patient died 11 months after the study treatment session because of the progression of another primary tumour and two additional patients died 26 and 27 months after treatment because of a hip fracture and progression of the melanoma, respectively. None of the cutaneous metastases categorized as CR relapsed during the 1-year follow-up period.

### 3.2. Adverse Events

**Figure 5:**
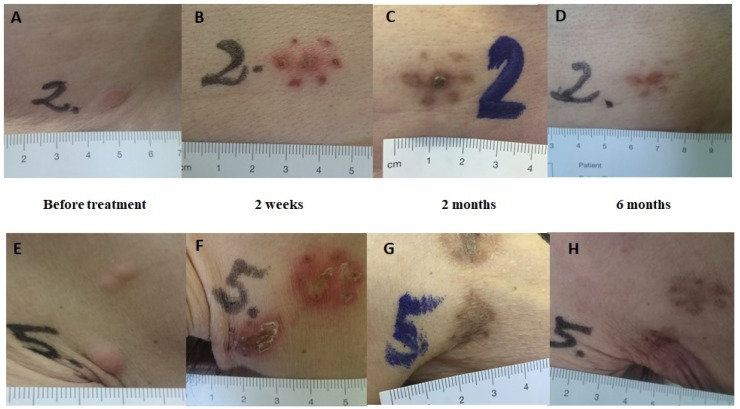
Clinical response. Clinical response after Ca-EP; clinical response after bleomycin-based ECT. The lesions are from patient no.2 with cutaneous metastases from breast cancer in the same region (trunk). Lesion no. 2: Ca-EP treated cutaneous metastasis. Lesion no. 5: bleomycin-based ECT treated cutaneous metastasis. (**A**,**E**) Before treatment. (**B**,**F**) Two weeks after treatment; typical crust appearance. (**C**,**G**) Two months after treatment; clear hyperpigmentation in the areas treated with calcium and bleomycin. (**D**,**H**) Six months after treatment; complete disappearance of metastases.

Serious adverse events were not observed. Ulceration and hyperpigmentation, both CTCA criteria grade I side effects, were seen after Ca-EP in two metastases each (2/18, 11%). After bleomycin-based ECT, ulceration was observed in 20% (3/15) and hyperpigmentation in 40% of the treated lesions (6/15) (Figure 5). The median NRS score before treatment was 2 (IQR = 2). Immediately after treatment the median NRS score was 2 (IQR = 9), 3 patients reported no pain (NRS: 0), 1 patient reported mild pain (NRS: 1–3) and 1 patient moderate pain (NRS: 4–6). Two patients, both having more than six metastases and who underwent biopsies, reported severe pain (NRS: 9–10). Six months after treatment the median NRS score was 2 (IQR = 4), with medium values of 2.5 (σ = 3.2016) for patients treated with Ca-EP and 4.5 (σ = 2.2913) for patients treated with ECT.

Six months after treatment, the QOL scores were equal to or higher than before treatment. The median QOL score was 70% (IQR = 10) before treatment and 80% (IQR = 10) at day 180. Three patients did not report a change in their quality of life during the 6 months after treatment. All 7 patients reported they would agree to repeat the treatment if necessary.

### 3.3. Delivered Current

There was no significant difference in the measured delivered current neither between the two treatment arms (*p* = 0.956) nor in non-irradiated metastases compared to irradiated metastases (*p* = 0.911). The median delivered current was 3.85 A (IQR = 3.75) in metastases treated with calcium and 4 A (IQR = 2.4375) with bleomycin. The median delivered current in metastases located in non-irradiated skin was 3.85 A (IQR = 3.45) and 3.95 A (IQR = 1.525) in metastases from previously irradiated skin. A total of 30 applications were used with a median value of 1 (range 1–6, IQR = 1) to the 18 randomized lesions treated with Ca-EP, and 22 applications with a median value of 1 (range 1–3, IQR = 1) to the 15 cutaneous metastases treated with bleomycin-based ECT. The median delivered current measured with linear electrodes in the two treatment arms (Ca-EP and bleomycin-based ECT) was 5 A (range 2.25–6.1, IQR = 2.1; 31 applications) and 2.5 A (range 1.4–4.2, IQR = 1.6; 21 applications) with hexagonal electrodes. In the Ca-EP arm the median delivered current was 2·5 A (range 1.4–4.2, IQR = 1.5; 11 applications) with hexagonal and 4 A (range 2.25–9, IQR = 3.5; 19 applications) with linear electrodes. In the bleomycin-based ECT group the median delivered current was 2.75 A (range 1.4–3.6, IQR = 1.7125; 10 applications) with hexagonal and 5.05 (range 4–6.1, IQR = 1.325; 12 applications) with linear electrodes (Table 2).

### 3.4. Discussion

Ca-EP is a novel anticancer treatment that has been used successfully with tumours exhibiting various histologies. Preclinical studies provided the first support for the efficacy across cancer histologies, as well as an explanation of the mechanisms of action [11,13,14,20,21,22,23,24,25,26,27]. The results of the first clinical trials suggested that Ca-EP is safe and efficient at the local level for tumours of different types, including cutaneous metastases from breast cancer and malignant melanoma and recurrent head and neck cancer [11,20]. Moreover, a case report showed that Ca-EP is able to initiate a systemic immune response and target untreated metastases in a patient suffering from malignant melanoma [28]. In the Ca-EP procedure, no chemotherapeutic drug is administered to the patient, and, therefore it is ideal in cases when chemotherapy is contradicted (e.g., severe lung function impairment, pregnancy etc.). Ca-EP is a simple and inexpensive cancer treatment and can lead to good cosmetic outcome.

We were encouraged to implement the second trial with Ca-EP on cutaneous metastases by the results of the first clinical studies and by the possibility that Ca-EP can be used in cases when bleomycin cannot be administered.

In our study, which included six patients with malignant melanoma and one patient with breast cancer, we demonstrated that Ca-EP is safe and effective in the treatment of small cutaneous metastases. Importantly, the CR seen clinically after Ca-EP was confirmed in our study by histology. The ORR for Ca-EP was lower (ORR = 33%, CR = 22%) than for bleomycin-based ECT (ORR = 53%, CR = 40%), but the differences were not significant for OR (*p* = 0.30) and for CR (*p* = 0.45). Our preset criteria for the non-inferiority for Ca-EP was proven. The first clinical trial performed in Denmark included seven patients: six patients with cutaneous metastases of breast cancer and one patient with malignant melanoma [11]. The results from this study had a similar tendency toward higher ORR and no significant difference between Ca-EP, 72% (with 66% CR), and ECT, 84% (with 68% CR). The first trial also proved non-inferiority for Ca-EP, and only mild adverse events were observed for both treatments, including ulcers in the treated area, similarly to our study. The higher response rates achieved by the Danish study could be explained by the different histotypes of the treated cutaneous metastases and also with the use of different electrodes. We have treated mainly malignant melanoma metastases (81.8%, *n* = 27), whereas the number of melanoma metastases was small (5.4%, *n* = 2) in the Danish study. The primary tumour characteristics of our study show that 89% (24/27) of the response evaluated melanoma metastases were BRAF-WT (wild-type or non-mutated). A recent study revealed that bleomycin-based ECT is more effective on BRAF mutated malignant melanoma cells in comparison with WT (non-mutated) melanoma cells [29]. These results could account for the lower response rates on both arms of the current study and require further investigation.

The other difference between the two trials were the electrodes used; only linear electrodes were used in the Danish, where as our study used mainly (63.6%) hexagonal electrodes. It is known that the electric field distribution is different between the linear (which have a smaller diameter) and hexagonal electrodes. Therefore, with linear electrodes field distribution is much more symmetrical with less cold spots. [30,31] Although a significant difference was not detected because of the small number of events, Ca-EP was more effective in our trial with linear electrodes, (*p* = 0.30). In preclinical studies significantly decreased ATP level and cell viability were observed by increasing the electric field from 0.8 to 1.0 kV/cm during Ca-EP [9,23]. These findings could explain the observed difference as the electric field was 1000 V/cm with the use of linear and 910 V/cm with hexagonal electrodes. Because of the small sample size of the current trial, further studies are needed to fully uncover the question.

There was no significant difference in the measured delivered current between the two treatment arms in the two finished studies. The difference in conductivity may be more relevant when treating large tumours.

Only grade I local adverse events were seen in both treatment arms in our study. Both ulceration and hyperpigmentation were observed more often after bleomycin-based ECT (20% and 40%) than after Ca-EP (both 11%). Our observations of Ca-EP skin toxicity were similar to those of the previous trial: only the tumour region was affected by the ulcer, whereas the surrounding normal skin was spared. In the Danish trial, none of the lesions treated with Ca-EP exhibited altered cutaneous pigmentation, which might be related to the exclusive use of linear electrodes.

The limitations of our study include a small number of enrolled metastases and the use of different electrodes.

## 4. Conclusions

Ca-EP proved to be safe and effective in eradicating tumours, and this conclusion was confirmed histologically. Our results are in agreement with the results of the first clinical trial on Ca-EP, which showed that Ca-EP was non-inferior to bleomycin-based ECT, and therefore Ca-EP should be considered as a feasible treatment for patients with cutaneous metastases for which other chemotherapeutic drugs are contraindicated.

The configuration of the electrode and the histotype may influence tumour response. Further studies are needed to establish a strong evidence base in the treatment of cutaneous metastases with Ca-EP.

## Figures and Tables

**Table 1:** Baseline demographic and clinical characteristics of the patients.

Patient	Sex Age (year)	Primary Tumour Characteristics	Location of Cutan Metastases	Number of Metastases Included/Evaluated	Years Since Diagnosis	Previous Therapy	Concomitant Treatment
1.	Male 76	pT3b, ALM, BRAF-WT	lower extr.	1/1	1	-	-
2.	Female 62	Breast: HER2-, ER+, PgR+	trunk	10/6	5.6	Epi-txt, Letrozole, mTORi	Letrozole
3.	Female 83	pT3b, NM, BRAF-WT, satellite met. Ing. sentinel: pos. BD: negative	lower extr.	9/6	7	adj. IFN, radiotherapy, ECT	-
4.	Female 49	pT3b, SSM	lower extr.	3/3	2	adj. IFN	-
5.	Female 83	pT3a, ALM, BRAF-WT Ing. sentinel: neg.	lower extr.	10/6	4.5	ECT	-
6.	Female 64	pT2a, SSM, BRAF-WT	lower extr.	6/6	2.75	-	-
7.	Male 73	pT3a, ALM, BRAF-WT	lower extr.	5/5	3.8	adj. IFN, radiotherapy	-
Total	5 Females, 2 Males mean: 70 (σ = 11.4891)	MM: 6, BRAF-WT: 5/6 Breast: 1	6 lower extr. 1 trunk	44/33	Mean 3.8 (σ = 1.9329)	various	

MM: malignant melanoma; ALM: acral lentiginous melanoma; extr.: extremity; NM: nodular melanoma; SSM: superficial spreading melanoma; BD: block dissection, BRAF WT: BRAF wild type; IFN: interferon; σ: standard deviation.

**Table 2:** Results of the current study in comparison with the study published in 2018.

Treatment Arm	Calcium-Electroporation			Bleomycin-Based Electrochemotherapy		
	Current Study	2018 Study	Total	Current Study	2018 Study	Total
**Lesion characteristics**						
Tumour size						
Median of the largest diameter, mm	6.5 (5–30)	9.5 (5–18)		7 (5–25)	11 (4–25)	
Tumour type						
Malignant melanoma	15	1	16	12	1	13
Breast cancer	3	17	20	3	18	21
Previously irradiated lesions, *n*	2	8	10	4	7	11
Location						
Lower extremity	15	4	19	12	4	16
Trunk	3	14	17	0	15	15
Upper extremity	0	0	0	3	0	3
Treatment						
Median doses (range), mL	0.085 (0.042–3.14)	0.24 (0.03–1.21)		0.132 (0.065–0.475)	0.21 (0.03–0.55)	
Median delivered current (range), A	3.85 (1.4–9)	3.4 (0.9–8.2)		4 (1.4–6.5)	2.8 (1–9.6)	
Median delivered current with linear electrodes (range), A	4 (2.25–9)	3.4 (0.9–8.3)		*5.05 (4–6.1)*	2.8 (1–9.6)	
Median delivered current with hexagonal electrodes (range), A	2.5 (1.4–4.2)	NA		*2.75 (1.4–3.6)*	NA	
Median number of applications (range), *n*	1 (1–6)	3 (1–7)		1 (1–3)	3 (1–7)	
Electrodes						
Linear	39% (7)	100% (18)		33% (5)	100% (19)	
Response (CR) for linear electrode subgroup	14% (1)	66% (12)		0	68% (13)	
Hexagonal	61% (11)	0		67% (10)	0	
Response (CR) for hexagonal electrodes subgroup	27% (3)	NA		60% (6)	NA	
Response						
Complete response, percent (*n*)	4	12	44.44% (16)	6	13	55.88% (19)
Partial response, percent (*n*)	2	1	8.33% (3)	2	3	14.7% (5)
Stable disease, percent (*n*)	6	3	25% (9)	5	0	14.7% (5)
Progressive disease, percent (*n*)	6	2	22.22% (8)	2	3	14.7% (5)
Adverse events						
Ulceration, percent (*n*)	2	7	25% (9)	3	13	47.05% (16)
Itch, percent (*n*)	0	1	2.77% (1)	0	5	14.7% (5)
Hyperpigmentation, percent (*n*)	2	0	5.55% (2)	6	5	32.35% (11)
Exuding, percent (*n*)	0	2	5.55% (2)	0	2	5.88% (2)

Current study: Evaluation of calcium electroporation for the treatment of cutaneous metastases; a double blinded randomized controlled phase II trial, Department of Dermatology and Allergology, University of Szeged (ClinicalTrials.gov: NCT03628417). 2018 study: Calcium electroporation for treatment of cutaneous metastases; a randomized double-blinded phase II study, comparing the effect of calcium electroporation with electrochemotherapy, Denmark [11].
